# Selenium maintains cytosolic Ca^2+ ^homeostasis and preserves germination rates of maize pollen under H_2_O_2_-induced oxidative stress

**DOI:** 10.1038/s41598-019-49760-3

**Published:** 2019-09-18

**Authors:** Alberto Marco Del Pino, Marcello Guiducci, Roberto D’Amato, Alessandro Di Michele, Giacomo Tosti, Alessandro Datti, Carlo Alberto Palmerini

**Affiliations:** 1 Department of Agricultural, Food, and Environmental Sciences, University of Perugia, 06121 Perugia, Italy; 2 Department of Physics and Geology, University of Perugia, 06123 Perugia, Italy

**Keywords:** Plant stress responses, Plant sciences

## Abstract

Selenium (Se) displays antioxidant properties that can be exploited, in plants, to counteract abiotic stresses caused by overly-produced reactive oxygen species (ROS). Here, we show that fertigation of maize crops with sodium selenate effectively protects pollen against oxidative stress. Pollen isolated from Se-treated plants (Se1) and untreated controls (Se0) was incubated *in vitro* with H_2_O_2_ to produce oxidative challenge. Given the impact of ROS on Ca^2+^ homeostasis and Ca^2+^-dependent signaling, cytosolic Ca^2+^ was measured to monitor cellular perturbations. We found that H_2_O_2_ disrupted Ca^2+^ homeostasis in Se0 pollen only, while Se1 samples were preserved. The same trend was observed when Se0 samples were treated with sodium selenate or Se-methionine, which recapitulated *in vitro* the protective capacity of Se-fertigation. Furthermore, we found that germination rates were much better retained in Se1 as compared to Se0 (46% vs 8%, respectively) after exposure to 20 mM H_2_O_2_. The same was observed with Se0 pollen treated with Se-methionine, which is the organic form of Se into which most fertigated sodium selenate converts in the plant. These results, together, show a close correlation between ROS, Ca^2+^ homeostasis and pollen fertility, and provide strong evidence that Se-fertigation is an excellent approach to preserve or enhance agricultural productivity.

## Introduction

Selenium (Se) is a microelement that, over the last decade, has gained much attention in disease prevention in light of its antioxidant properties^1,2^. However, studies also indicated that, as a dietary supplement, Se should be taken with caution based on toxic effects that may appear at higher concentrations^3^. Se-intake from the diet can largely vary based on differences in soil composition of the land where plants are grown and animals raised. In this regard, a critical factor is the particular capacity of a given plant to metabolically engage, and subsequently accumulate, Se in various forms^4,5^. Frequently, foliar or soil Se-fertilization is undertaken in agricultural crops in order to improve Se concentration in edible plants^6–10^.

Se is known as a powerful antioxidant capable of counteracting ROS-induced oxidative stress^3,11,12^ and, for this reason, can be considered a protective agent against abiotic perturbations that may be caused by changes of temperature, sources of nutrients, and concentration of salts, which, ultimately, can cause ROS levels to rise^13^. In plants, ROS are commonly produced by the aerobic energy system in cellular organelles such as chloroplasts, mitochondria and peroxisomes^14^. At low concentrations, ROS act as signal molecules that become involved in numerous biological processes such as vegetative growth and seed production in plants^11,12,15–19^. However, increased levels of ROS, in any of its chemical varieties, result in oxidative stress as a consequence of inadequate scavenging ability of the cell^13,20^.

Higher plants have a specialized, sexual reproduction system based on transport and delivery over greater distances of large amounts of male microgametophytes (i.e. pollen grain). In Angiosperms, upon landing on the right stigma, pollen granules quickly hydrate and begin to germinate. Timely germination of the male microgametophyte followed by rapid elongation of the pollen tube are both essential to ensure the sexual reproduction of plants^21,22^. To this end, ion exchanges mediate cell signaling events that guide the male microgametophyte towards the female egg^19^.

In particular, a number of studies pointed to the physiological role of Ca^2+^ as a secondary messenger in plant cells^23–27^. In the plasma membrane of the plant cell, several channels regulate Ca^2+^entry, however, despite the ion’s permeability, none of these channels appeared to be Ca^2+^ specific^17^. Ca^2+^ enters the cell through these channels and activates vertical cell growth^19^. Additionally, the formation of the pollen tube is Ca^2+^-dependent in the pollination process^19,25,28,29^.

ROS-mediated oxidative stress can cause infertility of pollen with obvious negative repercussions on plant reproduction and, in turn, agricultural production^14^. High concentrations of ROS can alter the molecular signals of the cell, including that mediated by cytosolic Ca^2+^. ROS act like agonists by either stimulating the mobilization of the ion from internal Ca^2+^ stores such as endoplasmic reticulum, Golgi vesicles and vacuoles^30^, or activating the Ca^2+^ entry from the extracellular medium, thereby disrupting the thousands-fold gradient between nanomolar and micromolar concentrations that the cell tightly maintains in the cytosolic and the extracellular space, respectively^25,31–36^. A large body of literature describes the interaction between ROS and Ca^2+^, however only scant information exists about the effect of either inorganic or organic Se in the relationship between ROS, Ca^2+^ signals and pollen germination^25,31,37^.

Here, we provide evidence that Se, under oxidative stress, can maintain cytosolic Ca^2+^ and preserve germination of corn pollen grains, suggesting that Se should be included in soil fertilizers to enhance the plants’ response to ROS formation. Furthermore, results point to a strong correlation between levels of cytosolic Ca^2+^ and the mechanistic events that underlie the germination process.

## Results

### Morphology of maize pollen grains

**Figure 1:**
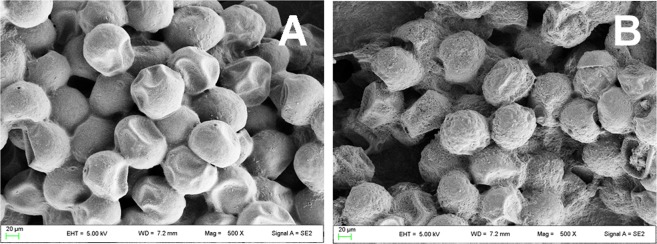
Morphology of pollen grains. Shown are images by Electron Scanning Microscopy (500X) of pollen grains in untreated controls (**A**) and Se-treated maize plants (**B**).

Initially, to assess any morphological alterations of pollen following treatment with Se, we generated images (500X) by Field Emission Scanning Electron Microscopy, as shown in Fig. 1. These revealed that the size and shape of Se-fertilized (Se1) and control (Se0) pollen grains were typical of maize species;^38^ however, Se1 samples displayed an especially rough, grainy appearance, which differed from a much smoother outer wall surface found in Se0 counterparts.

### Content and speciation of selenium in pollen and pollen supernatant

Selenium buildup in soil and subsequent uptake by crops, as well as distribution and accumulation of Se in different chemical forms can largely depend on fertilization protocols^5,6,8,9^. Thus, we decided to investigate abundance and chemical fate of Se-treated pollen. In untreated maize pollen, Se mainly accumulated in organic form such as Se-methionine (SeMet), which represented more than 95% of the organic pool, while Se(IV) accounted for about 2/3 of the total inorganic fraction (Table 1). On the other hand, Se-fertilization stimulated a marked increase (9.1-fold) in total selenium and, more particularly, its organic forms. Notably, while inorganic Se (Se[IV] and Se[VI]) showed, approximately, a 4-fold increase in Se1compared to Se0 samples, organic compounds such as SeCys, Se-Met, and MetSeCys displayed 8.8-,10.9- and >700-fold differences, respectively, with SeMet accounting for approximately 88% of the total amount of selenium (Table 1).

**Table 1:** Content and speciation of maize pollen Se in control (Se0) and Se-treated (Se1) maize plants.

	SeCys	MetSeCys	SeMet	Se (IV)	Se (VI)	Se tot
(μg /Kg FW ± SEM)						
Se0	12.6 ± 2.6	<0.1	198.3 ± 12.5	25.2 ± 1.4	11.7 ± 1.0	268.7 ± 46.1
Se1	111.4 ± 9.4	70.5 ± 2.7	2165.2 ± 42.8	87.6 ± 5.5	49.6 ± 3.8	2445.7 ± 177.8

Results are expressed as the means ± SEM (standard error of the mean) from three independent measurements. FW, fresh weight.

In addition, to further discriminate differences between Se0 and Se1 samples, we analyzed chemical contents in pollen supernatant, collected after the centrifugation step performed to isolate pollen grains. In Se1 supernatant, total protein and total Se levels were higher (35% and 53%), respectively than in Se0.

Since H_2_O_2_ was subsequently employed to challenge pollens with oxidative stress in order to test the antioxidant potential of Se treatment, we also analysed the supernatant obtained from pollen treated with 10 mM H_2_O_2_. In this regard, virtually identical results were found in both Se1 and Se0 samples (Table 2).

**Table 2:** Total Selenium and total protein concentrations in the supernatant obtained to isolate pollen of control (Se0) and Se-fertilised (Se1) maize plants, in the presence and absence of 10 mM H_2_O_2_.

	Se0	Se0 + H_2_O_2_	Se1	Se1 + H_2_O_2_
Proteins (µg/ml)	588.8 c ± 25.33	588.8 c ± 28.47	792.5 a ± 32.01	720.0 b ± 24.26
Selenium (μg/ Kg/FW)	1.9 c ± 0.21	1.2 d ± 0.42	2.9 a ± 0.43	2.7 b ± 0.33

Data show the means ± SEM obtained from three independent measurements. FW, fresh weight. Statistically significant differences are indicated by different letters, wheres identical letters highlight non-significant trends. SEM, standard error of the mean.

### Effect of oxidative stress on cytosolic calcium ([Ca^2+^]_cp_) in control and Se-fertilised pollen grains

With the overall objective of assessing the antioxidant potential of Se, we initially tested the impact of oxidative stress on levels of cytosolic Ca^2+^. To this end, 1–20 mM H_2_O_2_, a range previously found to be optimal for cell-based procedures^37^, was included with resuspended aliquots of pollen.

**Figure 2:**
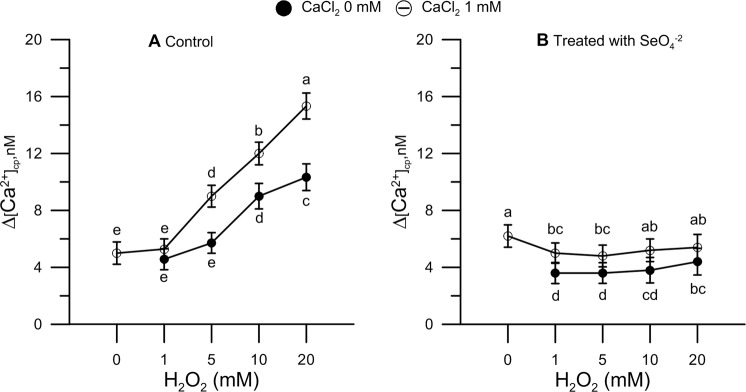
Oxidative stress and levels of cytosolic Ca^2+^ in pollen grains. The effect of H_2_O_2_ was tested using a range of concentration (1–20 mM), as shown. Cytosolic Ca^2+^ was measured in Se0 (panel A, control) and Se1 (panel B, treated with sodium selenate) pollen grains either in the presence (○) or absence (●) of 1 mM CaCl_2_ in the incubation medium to assess the extent of Ca^2+^ entry in pollen. Results are expressed as Δ[Ca^2+^]_cp_ (nM) to reflect signal changes detected between the start and the end of the fluorometric measurements, and represent means ± SEM from five independent tests. In both panels, at any given dose of H_2_O_2_, statistically significant differences between ○ and ● are indicated by different letters, wheres identical letters highlight non-significant trends. SEM, standard error of the mean.

Measurements performed with the FURA-2AM probe showed Δ[Ca^2+^]_cp_ to be in the 5–6 nM range in untreated samples. However, [Ca^2+^]_cp_ significantly increased in the presence of 5, 10, and 20 mM H_2_O_2_ (Fig. 2A).

To monitor the entry of Ca^2+^ from the extracellular space, we exogenously included 1 mM CaCl_2_ in the reaction mixture 250 s after the start of the fluorometric measurement (37). As shown in Fig. 2A, we found that Ca^2+^ entry was essentially negligible in the absence of H_2_O_2_ and up to concentrations of 1 mM H_2_O_2_. Instead, Ca^2+^ entry, ultimately leading to higher [Ca^2+^]_cp_, was evidently increased, in a dose-dependent fashion, with higher doses (5–20 mM) of H_2_O_2_ (Fig. 2A).

Conversely, in Se1 pollen, [Ca^2+^]_cp_ and Ca^2+^ entry were unaffected by H_2_O_2_ under the same conditions (Fig. 2B).

### Effects of sodium selenate and Se-methionine on ([Ca^2+^]_cp_) under H_2_O_2_-induced oxidative stress

To assess the effect of inorganic and organic Se under oxidative stress, we carried out analyses *in vitro* using untreated (Se0) maize pollen in the presence of increasing doses (1–15 μM) of sodium-selenate (SeO_4_^-2^) and Se-methionine (SeMet) in pollen incubation medium supplemented with 10 mM H_2_O_2_.

**Figure 3:**
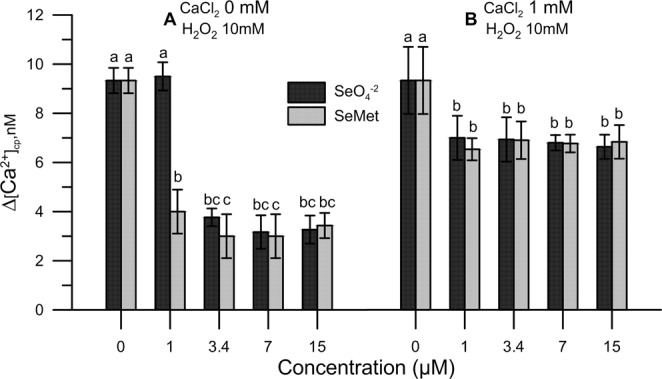
Effect of sodium selenate (SeO_4_^−2^) and selenium methionine (SeMet) on pollen grains subject to oxidative stress. In all instances, oxidative stress was induced with 10 mM H_2_O_2_. Changes in cytosolic Ca^2+^ were assessed in Se0 pollen exposed to SeO_4_^−2^ or SeMet employed at doses ranging from 1 to 15 μM. The addition of SeO_4_^−2^ or SeMet to the incubation medium was performed 50 s prior to the treatment with H_2_O_2_, after which fluorometric measurements were immediately started. CaCl_2_ (1 mM) was included (*right panel*) to assess the extent of Ca^2+^ entry. Data are expressed as means ± SEM from five independent tests. In both panels, at any given concentration of SeO_4_^−2^ or SeMet, statistically significant differences between paired columns are indicated by different letters, whereas identical letters highlight non-significant trends. SEM, standard error of the mean.

Pre-incubating the pollen suspension with SeO_4_^-2^ or SeMet mitigated the effect of 10 mM H_2_O_2_, which could not be detected any longer when SeO_4_^-2^ and SeMet doses achieved 3.4 μM and 1 μM, respectively (Fig. 3A). Additionally, pre-treatment with SeO_4_^-2^ or SeMet prevented the increase of Ca^2+^-entry when 1 mM CaCl_2_ was included in the medium (Fig. 3B).

For comparison purposes, we tested SeO_4_^-2^ and SeMet in the absence of H_2_O_2_ and, as expected, did not find any effects on both internal Ca^2+^ levels and Ca^2+^-entry (data not shown).

### Germination of maize pollen under oxidative stress

**Figure 4:**
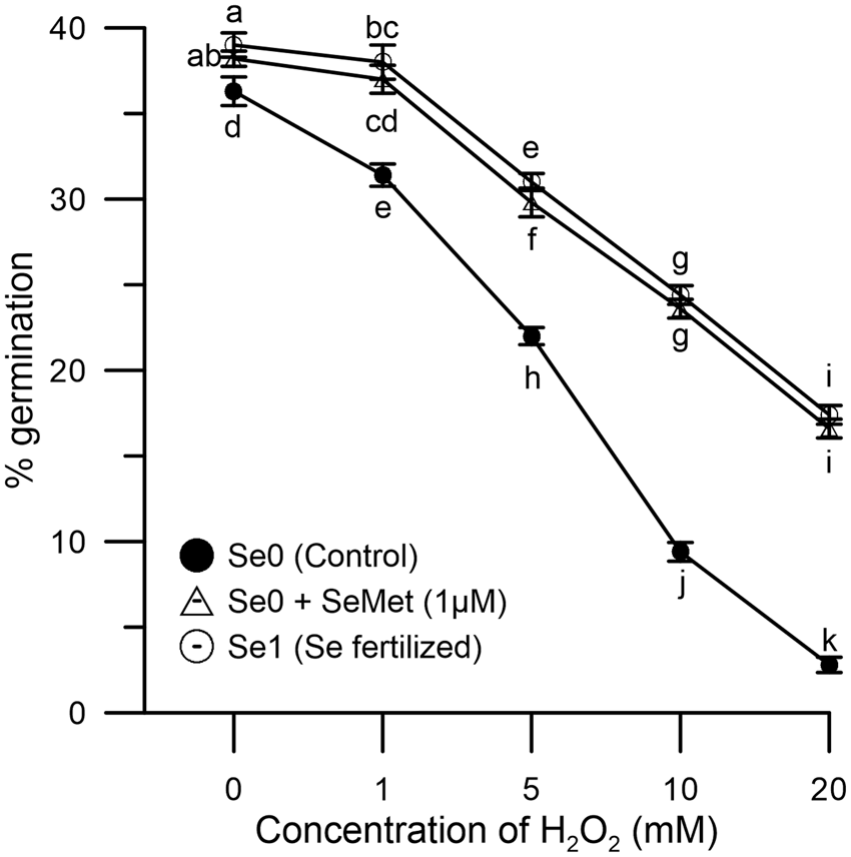
Germination rates under oxidative stress. Different concentrations of H_2_O_2_ (1-20 mM) were used to induce oxidative stress. The graph shows data obtained from Se0 (●, control), Se0 exposed to 1 μM SeMet (Δ), and Se1 (○) pollen. Tests were performed as described in the Materials and Methods. Germination was confirmed when the pollen tube was found to be longer than the pollen diameter. Results are reported as % of germinated pollen within a population of one hundred grains, and are expressed as means ± SEM from five independent tests, each of which included three technical replicates. As in Figs 3, 4, statistical significance of each set of data corresponding to a given dose of H_2_O_2_ is indicated by different letters. SEM, standard error of the mean.

Germination rates of maize pollen under normal conditions compared well with results obtained by different authors^39,40^. As shown in Fig. 4, in the absence of oxidative stress, pollen germination was similar in Se1(39% ± 1.1) and Se0 treated pollen (36% ± 0.8), whereas, in both pollen groups, H_2_O_2_ reduced rates in a dose-dependent fashion. However, the inhibitory effect of H_2_O_2_ was much more pronounced in Se0 than Se1 pollen, as germination curves at increasing H_2_O_2_ concentration progressively diverged. At the highest H_2_O_2_ concentration employed (20 mM), Se-treated pollen germinated at 18% ± 0.5 rate, which represented 46% efficiency in relation to normal conditions. Instead, the same amount of H_2_O_2_ almost abolished (3% rate, 8% efficiency) germination in untreated pollen. Interestingly, Se0 pollen exposed *in vitro* to 1 μM SeMet exhibited germination rates similar to Se1 (Fig. 4).

### Germination rates of control maize pollen grains (Se0) in the presence of SeO_4_^-2^and SeMet

**Figure 5:**
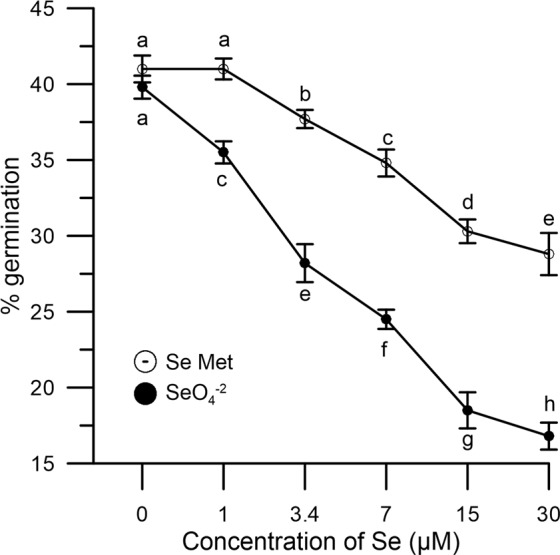
Effect of SeO_4_^−2^ and SeMet on germination rates. Se0 pollen was incubated with increasing concentrations (1–30 µM) of SeO_4_^-2^ (●) or SeMet (○). Results are expressed as % germination (as in Fig. 4) and represent the means ± SEM from five independent measurements (each of which supported by three technical replicates). Statistical significance of each set of data corresponding to a given dose of Se forms is indicated by different letters. SEM, standard error of the mean.

In the absence of oxidative stress, incubation of Se0 samples *in vitro* with increasing concentrations (1–30 μM) of SeO_4_^−2^ or SeMet, caused a dose-dependent reduction of germination ability. Figure 5 shows that the effect of the inorganic SeO_4_^−2^ form was slightly stronger than that displayed by organic SeMet.

## Discussion

Here, we show that pollen obtained from maize crops exposed to a Se-supplemented fertilization regimen has a superior capacity to counteract ROS-mediated effects that may negatively impact germination rates.

Our experimental setup implied field maize fertigation with sodium selenate, isolation of pollen from plants and, subsequently, application of H_2_O_2_ in pollen preparations to raise oxidative stress *in vitro*. Conditions related to fertigation, and the setup of the cellular oxidative model were previously established (shown in refs^9,37^., respectively).

Pollen samples, from plants either treated (Se1) or untreated (Se0) with sodium selenate, were used to measure cytosolic levels of Ca^2+^ in the presence and absence of H_2_O_2_. Cytosolic Ca^2+^ plays a major role as a second messenger, and alterations of Ca^2+^ homeostasis may prompt molecular switches in the regulation and signalling networks of the pollen tube^24,29^. Furthermore, germinated pollen grains and elongated pollen tubes require an internal Ca^2+^ gradient that is maintained by an extracellular ion supply^12,19,25^. Thus, in light of the well-documented, two-way relationship between ROS, which can modulate calcium-dependent cellular networks, and calcium signaling, which plays a key role in ROS assembly^31–36^, we decided to use cytosolic Ca^2+^ as the end-point for monitoring perturbations caused by oxidative stress.

We found that H_2_O_2_-induced oxidative stress caused a significant alteration of Ca^2+^ homeostasis, shown by an increase in cytosolic Ca^2+^ and a markedly enhanced Ca^2+^ entry when 1 mM CaCl_2_ was exogenously added to the pollen resuspension medium. By contrast, Ca^2+^ homeostasis was fully maintained in both Se1 pollen and Se0 pollen (i.e., pollen from plants not fertigated with sodium selenate) exposed to either sodium selenate or Se-methionine *in vitro* for 50 s prior to the addition of H_2_O_2_ and, immediately after, the start of measurements. Given the short incubation times, it is possible to rule out, at least *in vitro*, the implication of any metabolic mechanism and conclude that Se, in either chemical form, acts basically as a mere ROS scavenger that ultimately prevents ROS-mediated dysfunction of Ca^2+^ channels. Our results, however, indicate that the pollen obtained from Se-fertilized maize plants, as compared to Se0 controls, consisted of >95% organic Se, thereby suggesting that the inorganic form fertigated into crops was metabolically, and almost quantitatively, converted in Se-methionine (88%) and other organic forms such as SeCys and MetSeCys.

The capacity of Se1 pollen to tolerate oxidative stress (46% efficiency in Se1 vs 8% in Se0 with 20 mM H_2_O_2_) is especially important for agricultural productivity, based on multiple abiotic factors that can ultimately lead to an excess of ROS production^13,14,20^, and the observation made by other groups showing that abiotic stresses of different nature may inevitably lead to excessive ROS accumulation and, consequently, pollen sterility^14^.

Additionally, it must be noted that Se-fertigation, despite resulting in a total content of Se in pollen that was approximately 10-fold that of untreated crops, did not impact germination rates in the absence of oxidative stress. Rather, robust germination rates (39% vs 36% in Se1 and Se0, respectively), that were found to be within a range previously reported in the literature^39,40^, ruled out toxicity concerns. However, as shown in Fig. 5, higher doses (>1 μM) of inorganic or organic Se may ultimately impact fertility, although a comparison between data from Se-fertigation and *in vitro* Se treatments should be cautiously taken, given the capacity of fertilized plants to adapt to Se over a much longer term. In any case, any Se-fertigation protocol must be thoroughly validated considering the multiple environmental factors, both biotic and abiotic, that may influence agriculture.

Biochemical elucidation of the effects of Se in pollen are likely required in order to optimize conditions and address possible unexpected events or, perhaps, uncover mechanistic events that could further improve germination rates. For example, it will be interesting to understand why the surface of Se1 pollen is morphologically different compared to normal Se0 samples, as shown by Field Emission Scanning Electron Microscopy. The rough aspect of Se1 pollen may relate to a higher permeability of the outer wall to proteins and Se itself (reported in Table 1), suggesting the possibility of thinner and/or more fragile wall layer(s).

In conclusion, we provide strong evidence, for the first time, about the beneficial effects of Se-fertigation in maize pollen. Additionally, we demonstrate that measurement of cytosolic Ca^2+^ is an easy, quick determination that can be used to monitor the occurrence of oxidative stress and the efficacy of antioxidative measures.

## Materials and methods

### Reagents

FURA 2-AM (CAS# 108964-32-5), PBS (Phosphate Buffered Saline), Triton X-100, EGTA, sodium selenate (Na_2_SeO_4_), selenium methionine, hydrogen peroxide (H_2_O_2_), sodium chloride (NaCl), potassium chloride (KCl), magnesium chloride (MgCl_2_), glucose, Hepes, and dimethyl sulfoxide (DMSO), were acquired from SigmaAldrich Italy. Any other chemicals and reagents were of the highest quality, and obtained from reputable, commercial sources.

### Site description, treatments and experimental design

Maize (*Zea mays L*., hybrid ISH302v from ISTA, Agroalimentare Sud S.p.A., Italy) was grown in 2018 at the Field-Lab of the University of Perugia located in Papiano (43°N-12°E, elevation 541 ft.), in a clay-loam Fluventic Haplustepts soil^41^ featuring 10.3 g kg^−1^ of total organic C, 250 µg kg^−1^ of total Se and a pH of 7.75.

Crop was sown on April 24^th^, 2018 in single rows with 0.75 m spacing in between. The emergence process of the plants proceeded as expected; density was 6.90 ± 0.13 m^−2^ at the time of maize V2 stage^42^.

At the time of seeding, fertilizers were applied with broadcast distribution of urea (150 kg N ha^−1^). To prevent crops from the occurrence of weeds, we employed pre-emergence herbicides such as Terbuthylazine 17.4% + S-Metolachlor 28.9% (4.0 L ha^−1^) and hand hoeing.

To control evapotranspiration, crops were irrigated using drip lines (streamline plus 16080, Netafim srl, Italy) placed on soil surface beside each seeding row.

Experimental procedures, aimed at evaluating fertilization conditions, implied block randomization with four replicates. Se treatments included i) no external Se input (Se0, used as the reference), and ii) Se fertilization (Se1). In Se1, 100 g sodium selenate ha^−1^ (1449 mg plant^−1^) was applied by fertigation on June 25^th^ 2018. Elementary plots consisted of 24 maize rows, each of which was 20 m long.

### Maize pollen collection

Pollen was sampled at maize silk (R1 Stage) on June 3^rd^ 2018. Male inflorescences were shaken and pollen rain harvested with a steel funnel (20 cm diameter) outfitted with a steel filter (0.5 mm) to reduce debris (chaff and stamens) and keep insects out.

Pollen harvesting was carried out early in the morning with stable weather conditions (wind speed <1.9 ms-1; air temperature ranging between 26.7 and 28.7 °C, and relative humidity between 69% and 58%).

To minimize cross contamination, sampling of each replicate was performed by a dedicated group of technicians, and harvesting devices were thoroughly and consistently cleaned after each sampling procedure.

Within ten minutes from collection, pollen from each plot was cleaned up by hand, placed in polypropylene tubes wrapped with aluminum foil, and immediately transferred into a refrigerated container (5 °C).

### Pollen preparations and measurement of Selenium and total protein content

Fresh pollen (200 mg/sample) was resuspended in PBS (8 ml) for 48 h in the dark, after which pollen supernatant was obtained by centrifugation (2000 g, 3 min). Both pollen grains and the correspondent supernatant were used to determine total selenium content. To this end, we employed a previously described protocol^37,43^, with minor modifications related to the biological material used, as previously described. Briefly, pollen (0.5 g/sample) was microwave-digested (ETHOS One high-performance microwave digestion system; Milestone Inc., Italy) with 8 mL of nitric acid (65%w/w) and 2 mL of hydrogen peroxide (30%w/w), after which digests were diluted with 20 mL water, passed through 0.45 μm filters, and tested by graphite furnace atomic absorption spectroscopy in a Shimadzu AA-6800 apparatus (GF-AAS; GFA-EX7, Shimadzu Corp., Japan) equipped with deuterium lamp background correction and a matrix modifier (Pd(NO_3_)_2_, 0.5 mol L^−1^ in HNO_3_. Determinations were carried out in triplicate.

Total protein content in supernatant was assessed by the Bradford’s method, with bovine serum albumin used as the standard^44^.

### Selenium speciation in pollen by HPLC ICPMS

The procedure employed to breakdown Selenium content in its different chemical forms was already described by Fontanella *et al*. in^43^. Briefly, fresh pollen (0.25 g/sample in 10 mL PBS) was treated with pronase (20 mg), sonicated for 2 min, and stirred in a waterbath at 37 °C for 4 h. Samples were then centrifuged, and the supernatant 0.22 μm- filtered. Se analyses were conducted by anion-exchange HPLC using an Agilent 1100 instrument equipped with a Hamilton PRP-X100, 250 × 4.6 mm column. Standard solutions (1, 5, 10, and 20 μg L^−1^) of inorganic [i.e., selenite, (SeO_3_^−2^) and selenate, (SeO_4_^−2^)] and organic [i.e., selenocysteine, (SeCys); Se-(methyl)selenocysteine, (SeMeSeCys); selenomethionine, (SeMet)] forms of Se were prepared using ultrapure (18.2 MΩ cm) water^43^.

### Images of maize pollen grains by Electron Scanning Microscopy

The morphology of the pollen samples was examined at 500x by Field Emission Gun Electron Scanning Microscopy using a LEO 1525 Gemini workstation (ZEISS) following Chromium metallization.

### Sample preparation, experimental oxidative stress, and measurement of cytosolic Ca^2+^

Pollen samples (100 mg), stored in the dark at 5 °C, were initially resuspended in 10 ml PBS, and hydrated for 2 days at 25 °C. Hydrated pollen was then collected by centrifugation (1000 g, 4 min), and resuspended in 2 mL Ca^2+^-free HBSS buffer composed of 120 mM NaCl, 5.0 mM KCl, MgCl_2_ 1 mM, 5 mM glucose, 25 mM Hepes, pH 7.4.

Oxidative stress was induced with variable doses (between 1 and 20 mM, shown in the Results) of H_2_O_2_, which was directly added to pollen suspensions immediately prior to measurements.

Intracellular levels of calcium were measured using the fluorometric FURA-2AM indicator dye according to a protocol previously described by our group^37^. Samples were incubated with FURA-2AM (2 μL of a 2 mM solution in DMSO) for 120 min, then centrifuged (1000 g, 4 min). Pollens were then resuspended in ~10 mL of Ca^2+^-free HBSS, supplemented with 0.1 mM EGTA to reduce background signal caused by contaminating ions, to obtain a suspension containing 10^6^ pollen grains/mL.

Fluorometric measurements were performed in a Perkin-Elmer LS 50 B spectrofluorometer (ex. 340 and 380 nm by dual-view wavelength splitter, em. 510 nm) with a 10 nm and a 7.5 nm slit width in the excitation and emission windows, respectively. Signals were taken after 300–350 s, a time range previously found to be optimal^37^. The signal ratio 340–380/510 nm was finally used to determine concentrations.

To this end, measurements were performed in two steps: initially, without calcium in the incubation medium and, 250 s later, in the presence of 1 mM CaCl_2_ to evaluate the accumulation in the cell of the extracellular ion^36^. Cytosolic calcium concentrations were calculated as reported by Grynkiewicz^45^.

### Pollen germination studies

Fresh pollen samples from each plot were hydrated in a humid chamber at room temperature for 30 min^46^, and then transferred to 6-well culture Corning plates (1 mg of pollen per plate) containing 3 mL of an agar-solidified growing medium composed of 1.2% agar, 10%, sucrose, 0.03% boric acid and 0.15% calcium chloride (pH 5.5)^39^.

Pollen suspensions were incubated for 24–48 h in a growth chamber at 27 °C with gentle shaking to ensure homogeneous distribution of the samples in the wells. To induce oxidative stress, H_2_O_2_ was directly dispensed in the culture wells.

Germinated and non-germinated pollen grains were counted under a 10X magnification microscope. Germination rates were calculated based on three replicates, each of which consisted of one hundred grains. Germination of grains was confirmed when the pollen tube had grown longer than the grain’s diameter^39^.

### Statistical analysis

Statistical evaluations were performed using the GraphPad Prism 6.03 software. Variance assessments included homogeneity analysis by the Levene’s test, and the normality test by D’Agostino-Pearson. Significance of differences was assessed by the Fisher’s least significant differences test. Differences with p < 0.05 were considered statistically significant.

## Data Availability

The Authors confirm that the dataset generated and used to compile this manuscript can be disclosed and made available by the corresponding author upon request.
